# Differences between Male and Female Consumers of Complementary and Alternative Medicine in a National US Population: A Secondary Analysis of 2012 NIHS Data

**DOI:** 10.1155/2015/413173

**Published:** 2015-03-11

**Authors:** Yan Zhang, Matthew J. Leach, Helen Hall, Tobias Sundberg, Lesley Ward, David Sibbritt, Jon Adams

**Affiliations:** ^1^Division of Health Services Research, Department of Family and Community Medicine, Laura W. Bush Institute for Women's Health, Texas Tech University Health Sciences Center, 3601 4th Street, Stop 8143, Lubbock, TX 79430, USA; ^2^International Complementary Medicine Research Leadership and Capacity Building Program, Australian Research Centre in Complementary and Integrative Medicine (ARCCIM), University of Technology, Sydney, NSW 2006, Australia; ^3^School of Nursing & Midwifery, University of South Australia, North Terrace, Adelaide, SA 5000, Australia; ^4^Faculty of Medicine, Nursing and Health Sciences, Monash University, Frankston, VIC 3199, Australia; ^5^Department of Neurobiology, Care Sciences and Society, Karolinska Institute, 141 83 Huddinge, Sweden; ^6^Department of Medicine, Dunedin School of Medicine, University of Otago, Dunedin 9054, New Zealand; ^7^Faculty of Health, University of Technology, Sydney, Building 10, Level 7, Room 232, 235-253 Jones Street, Ultimo, NSW 2007, Australia

## Abstract

We examined the National Health Interview Survey (NHIS) 2012 to explore how US adult consumers of CAM differ by gender in terms of their sociodemographic characteristics, current health conditions, and perceived benefits of CAM. All individuals who completed the adults core interviews (*N* = 34,525) were included. CAM use, major sociodemographic variables, perceived benefits of using CAM, and top ten reported health conditions for which CAM was used were selected and analyzed by Stata. Findings revealed that 29.6% (*n* = 10,181) reported having used at least one form of CAM in the previous 12 months. Compared to male CAM users, female CAM users were more likely to have a bachelor degree, to be divorced/separated or widowed, and less likely to earn $75,000 or more. Back pain/problem was the most common problem reported by both male and female CAM users (32.2% and 22.6%, resp.). A higher proportion of female CAM users reported using CAM for perceived benefits such as general wellness or general disease prevention. This paper provides foundation information regarding gender differences in CAM use and is a platform for further in-depth examination into how and why males and females differ in their reasons for CAM use.

## 1. Introduction

The use of complementary and alternative medicine (CAM) amongst adults is substantial in both the United States (USA) [1–4] and internationally [5–7]. In the USA, the use of CAM among adults increased considerably during the 1990s and has remained at a relatively stable rate (36–38%) over the past ten years [3, 4, 8]. A number of sociodemographic factors, including gender, ethnicity, and income, have been associated with an increased prevalence of CAM use [2, 3, 8].

A 2010 review of surveys investigating CAM use among community-based adults indicated an association between CAM use and gender, with women more likely than men to use CAM [5]. This corroborates the findings of previous National Health Interview Survey (NHIS) reports [3, 4]. A higher use of CAM among females is also evident in a number of clinical populations, including patients with cancer [9], acute coronary syndrome [10], and diabetes [11]. A US survey of Asian-American subgroups found that, contrary to findings in the broader population, CAM use may be lower among women compared to men within certain ethnicities [12]. A recent study in Norway also revealed that the relationship between demographics and CAM use differed significantly between men and women on age, household income, and marital status [13].

Gender differences in social determinants of health and illness, as well as health care decision-making, have been explored by various researchers over the past two decades [14–16]. There is clear evidence that disparities exist between men and women regarding the diagnoses and treatment of health conditions [17]. Further exploration of these disparities may provide additional insight into the gender inequities facing consumers in the current health care system, knowledge that is critical to understanding the health care needs of both women and men in the future.

To date, few have examined in depth the factors that differentiate male and female consumers of CAM. In response to this significant research gap, this paper reports the first focused analysis of gender differences in CAM use amongst US adults. Using data from the 2012 NHIS, this study specifically aimed to explore how US adult consumers of CAM differ by gender in terms of their sociodemographic characteristics, current health conditions, and perceived benefits of CAM.

## 2. Methods

### 2.1. Data Sources

This study is a secondary analysis of 2012 National Health Interview Survey data. NHIS is a cross-sectional household interview survey conducted periodically by Centers for Disease Control and Prevention's (CDC) National Center for Health Statistics (NCHS). The target population for the NHIS is the civilian noninstitutionalized population of the United States. The core questionnaires provide information on demographics, health status, health behaviors, and health care access and utilization. Supplemental questions on CAM use (i.e., Adult CAM Supplement) are collected every five years on randomly selected members of a household, including one adult (18 years or older) and one child (0–17 years old). The total household response rate for 2012 was 77.6%. The interviewed sample consisted of 42,366 households, which yielded 108,131 persons in 43,345 families, including 34,525 persons being 18 years of age and older. The conditional response rate for the sample adult component was 79.7% (i.e., the number of completed sample adult interviews [*n* = 34,525]/the number of eligible sample adults [*n* = 43,323]). Further details of the NHIS sample are reported elsewhere [18].

In the 2012 NHIS, the Adult CAM Supplement collected information from sample adults regarding their use of 18 nonconventional health care practices, including acupuncture, Ayurveda, biofeedback, chelation therapy, chiropractic or osteopathic manipulation, craniosacral therapy, energy healing therapy, hypnosis, massage, naturopathy, traditional healing, movement therapy (Pilates/Trager psychophysical integration/Feldenkrais), herbal and nonvitamin supplementation, vitamin and mineral supplementation, homeopathy, special diets, yoga/tai chi/qi gong, and relaxation techniques (meditation/guided imagery/progressive relaxation). While some questions were asked of each health care practice, other questions were asked only for the top three modalities deemed by the respondent to be the most important to their health [18]. For the “top three question series,” all modalities were included except for Ayurveda, chelation therapy, and vitamin and mineral supplementation. These three therapies were excluded due to either very low or high prevalence [18]. The topics covered in the top three question series, and also included in our study, include the following: reasons for using the modality; whether the modality motivated the respondent to engage in other selected health behaviors; outcomes associated with using the modality; whether the modality was used to treat a specific health problem or condition, and, if so, what health problems or conditions were treated, and for which one of the health problems or conditions the modality was used the most [18]. The current analysis utilized the Sample Adult File and Adult Alternative Medicine 2012 datasets published online by the CDC [19].

### 2.2. Measures

Based on the study objectives, CAM use, major sociodemographic variables, and perceived benefits of using CAM, as well as the most popular health conditions for which CAM was used, were selected as the main variables of the study, each of which are defined below.

*CAM Use*. Based on the counted number of CAM modalities used in the past 12 months respondents with a zero count were defined as non-CAM users, while those who reported the use of at least one CAM modality were defined as CAM users. 

*Sociodemographic Measures.* These included the following variables: sex (male and female), age (in years), race/ethnicity (Hispanic-White, non-Hispanic White, non-Hispanic Black, non-Hispanic Asian, and non-Hispanic other), educational attainment (less than high school, high school graduates and/or with some certified degree after high school, bachelor degree, and master degree or higher), marital status (currently married or living together but not married), and region of residence (Northeast, North Central/Midwest, South, and West). 

*Perceived Benefits of CAM.* These were captured under three distinct sets of yes/no survey questions regarding the first of the three top therapy modalities deemed by the respondent to be most important to their health, including the following:

- reasons for using CAM for the first top therapy (i.e., general wellness or general disease prevention, improving energy, improving immune function, improving athletic or sports performance, and improving memory or concentration);
- motivations for using CAM for the first top therapy (i.e., eating healthier, eating more organic foods, cutting back on or stop drinking alcohol, and doing exercise more regularly);
- outcomes of CAM use for the first top therapy (i.e., sense of control over one's health, reduced stress level or relaxation, better sleep, feeling better emotionally, coping with health problems easier, improved overall health and feeling better, improved relationships with others, and improved attendance at job or school).

Two questions regarding the helpfulness of CAM use were also included under perceived benefits. The two questions asked how much the first top therapy helped with the most important reasons for CAM use and with the specific health problems, respectively. Responses to these two questions were a great deal, some, only a little, or not at all. 

*Health Conditions*. Participants were asked whether a CAM modality was used to treat any of 86 specific health problems or conditions. For the purpose of this study, only the top ten specific health problems/conditions were compared between men and women.

### 2.3. Statistical Analyses

Analyses were performed using Stata 9.0 (Stata Statistical Software: Release 9. College Station, TX: StataCorp LP). All analyses used the NHIS Sample Adult Weight—Final Annual (WTFA_SA) including design, ratio, nonresponse, and poststratification adjustments for sample adults [18]. Stata survey commands were used for the complex survey sample design. Overall analysis included examination of the weighted comparison (Chi-Square and Independent *t*-test) between male and female CAM users in terms of their sociodemographic profile, perceived benefits of CAM use, and the top ten conditions that CAM was used for. Weighted logistic regression was performed on all adults to determine which sociodemographic variables were significantly associated with CAM use. Due to the large sample size, statistical significance was set at 0.01.

## 3. Results

In NHIS 2012, 34,325 adults were included in the Adult CAM Supplement subset. Of these, 29.6% (*n* = 10,181) reported having used at least one form of CAM in the previous 12 months. The sociodemographic characteristics of non-CAM and CAM users are presented in Table 1. Compared to non-CAM users, CAM users were more likely to be female, reside in the Midwestern or Western USA, be non-Hispanic White, have a bachelor degree or higher, have higher personal earnings, be married or living with a partner, and have greater family spending on medical care.

Of the CAM users, nearly 60% were women. In addition, female CAM users were significantly more likely than male CAM users to use more than one CAM modality (male: 1.7 ± 0.02 CAMs used; female: 2.0 ± 0.02 CAMs used; *P* < 0.001). Compared to male CAM users, female CAM users were also more likely to have a bachelor degree, and be divorced/separated or widowed, and be less likely to earn $75,000 or more. There were no statistically significant differences between male and female CAM users with respect to age, region, race/ethnicity, and family medical expense.

Using the total sample, logistic regression revealed that women were about three times more likely than men to use CAM (OR = 2.8; 95% CI: 2.5, 3.0), while race/ethnicity, education, personal earnings in the past year, family medical expenses, and marital status were all associated with CAM use (Table 2). Stratifying by gender, logistic regression models suggested different sociodemographic profiles of male and female CAM users (Table 2). For males, those who lived in the West were nearly twice as likely to use CAM as those who lived in the Northeast (OR = 1.78; 95% CI: 1.41, 2.26). For females, compared to those living in the Northeast, those living in both the Midwest (OR = 1.69; 95% CI: 1.37, 2.05) and the West (OR = 3.21; 95% CI: 2.61, 3.93) were more likely to use CAM; those living in the South (OR = 0.63; 95% CI: 0.52, 0.78) were less likely to use CAM. Compared to their Hispanic counterparts, all other races/ethnicities except non-Hispanic Asian men were significantly more likely to use CAM, while only non-Hispanic White women were more likely to use CAM.

Higher education was associated with CAM use among both men and women, with the exception of an insignificant difference when comparing high school to less than high school among women. When compared to no family medical expenses, higher expenses in women were not associated with a higher likelihood of CAM use, but expenses between $3000 and $4999 were associated with more CAM use in men (OR = 1.79; 95% CI: 1.24, 2.60). Both widowed men (OR = 3.06; 95% CI: 2.41, 3.89) and women (OR = 2.85; 95% CI: 2.39, 3.39), as well as divorced or separated women (OR = 2.06; 95% CI: 1.75, 2.41), were more likely to use CAM compared to men/women who were married and living together.

**Figure 1:**
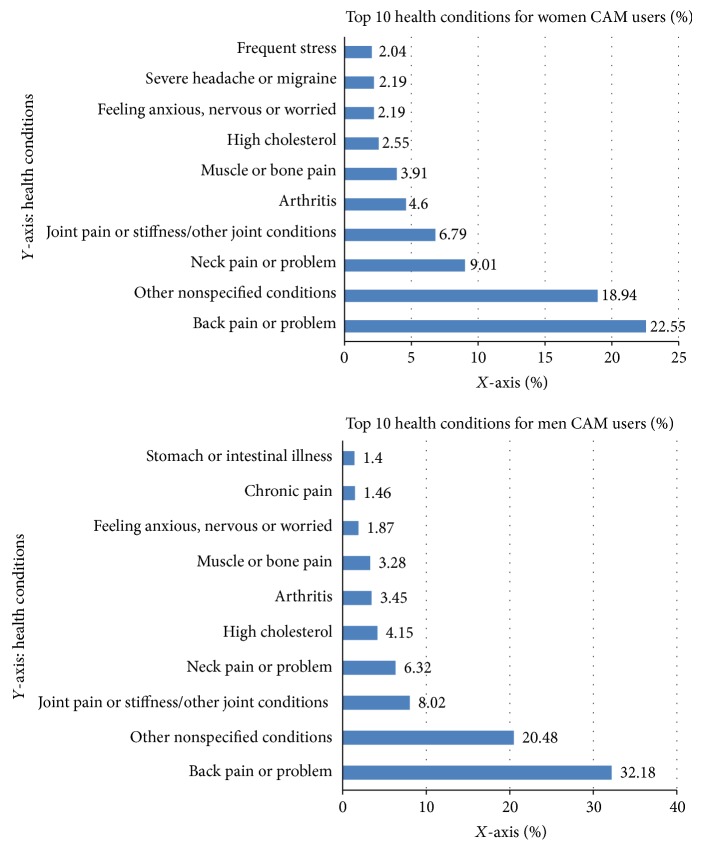
Top 10 health problems that men and women used CAM for.

Figure 1 presents the top ten most frequently reported health specific problems that CAM was used for. The top eight health conditions reported by male and female CAM users were very similar, although they did vary in order. Back pain/problem was the most common problem reported by both male and female CAM users (32.2% and 22.6%, resp.). Neck pain/problem and joint pain stiffness were also common problems reported amongst male and female CAM users. The last two of the top ten conditions reported were chronic pain (1.46%) and stomach or intestinal illness (1.4%) by male CAM users and severe headache or migraine (2.19%) and frequent stress (2.04%) by female CAM users.

The perceived benefits of CAM use, as reported by the men and women in our analyses, are presented in Table 3. There were no significant differences found between male and female CAM users in the use of CAM to improve memory or concentration, to cut back on or stop drinking alcohol or smoking cigarettes, to improve relationships with others, or to improve attendance at job or school, or to help a specific health condition. A higher proportion of female CAM users reported using CAM for general wellness or general disease prevention, to improve energy, to eat healthier or to eat more organic foods, to exercise more regularly, to give a sense of control over their health, and to reduce stress levels or relaxation, for better sleep, and for feeling better emotionally, to cope with health problems easier, or to improve overall health and feeling better. Also, a higher proportion of female CAM users rated the first of their top three CAM therapies as helping a great deal with the most important reasons for CAM use and with specific health problems. Compared to women who use CAM, male CAM users only reported higher CAM use to improve athletic or sports performance.

## 4. Discussion

This study has interrogated data from the Adult CAM Supplement of the 2012 NHIS in order to further our understanding of gender differences with respect to CAM use. Specifically, the study has gained new insights into how male and female CAM users differ in their sociodemographic characteristics, reasons, and motivations for CAM use and the health conditions for which CAM is used, shedding new light on the profile of CAM users in the USA.

### 4.1. The Sociodemographic Profiles

Our findings indicate that, compared to non-CAM users, users of CAM are more likely to be women, to have higher education and earnings, to be divorced or widowed, and not to reside in the South of the USA. Women, representing the majority (60%) of CAM consumers, were three times more likely to use CAM than men. This supports previous NHIS reports of CAM use in 2002 and 2007 [3, 4], as well as recent international findings from Europe and Australia [7, 20, 21].

In addition to the above-mentioned factors differentiating CAM users from nonusers, several elements clearly distinguished male consumers from female consumers. Firstly, personal income level differs between male and female CAM users, although it is not different between CAM and non-CAM users. Male CAM users, for instance, were more likely to earn a higher income (US$35,000 or more per annum) than female users. This observation is in line with the findings from a recent Norwegian study [13]. Previous studies have also explored whether or not income plays a role in CAM use [2, 5, 22, 23]. Our findings suggest that gender, income, and other socioeconomic statuses such as employment status may have a combined impact on CAM use. For instance, our study showed that female CAM users were more likely not to have worked in the past year (30.9%) compared to male CAM users (21.7%). The reason for this disparity is not entirely clear. Additional data on household income, employment status, family arrangement, and health insurance status could provide further insight into this issue.

Secondly, marital status appears to have a different impact on male and female CAM users. While previous studies suggest that mutual support of married individuals may promote greater CAM use among married ones than among divorced counterparts [24–26], our findings reveal the contrary and show that male CAM users are more likely to be married compared to female CAM users. It is reported that single persons may have more time to focus on relaxation CAM modality such as yoga or other mind-body exercise than married couples who have other household commitments [27]. This theory may also be applied to married women as they are known to take on disproportionally more household work than married men [28]. Another explanation for this gender difference is that perhaps married men are to some degree positively influenced by their female partners to use CAM. Indeed, local social networks (such as being advised or recommended by family, friends, and colleagues) have been recognized previously as being influential in decision-making regarding CAM use [29, 30].

Thirdly, our analyses revealed that male and female CAM users may differ by race/ethnicity. Our findings showed that all ethnicities except non-Hispanic Asian in men and only non-Hispanic White in women were significantly more likely to use CAM. On a broader level this corresponds to previous NHIS findings that have reported Asian adults as being generally less likely to use CAM compared to White, native, or American-Indian adults [3]. Notwithstanding, a survey of an adult US Californian population, notably a prominent US region for CAM use, indicated that the use of CAM was significantly higher among Asian-Americans compared to national prevalence data [12]. Hence, it may be important to acknowledge that CAM use in the US may vary by region and by ethnic subgroups.

### 4.2. The Perceived Benefits

Of all the reasons, motivations, and outcomes surveyed, a significant differential between women and men was identified. Relative to men, women were motivated to use CAM for a number of reasons (e.g., general wellness or general disease prevention, improving energy, and improving immune function), all of which related to a need to improve one's health and well-being. To some extent, this finding is not surprising as women are generally more likely than men to utilize preventative health care services [31], to seek health information for both illness and wellness [32], and to have greater health care needs [33].

Male CAM users in our study, on the other hand, were more likely to use CAM to improve athletic or sports performance. This finding is consistent with an earlier survey in New York that more male than female reported the use of CAM to enhance performance [34]. According to Atkinson [35], part of the motivation for men using CAM for athletic purposes relates to the pursuit for masculinity or the need to reclaim a lost sense of masculinity. As Atkinson so eloquently states, men may be using “‘scientifically designed' sports-supplement products to solve social and psychological (psychogenic) anxieties” [35]. It has previously been identified that men's understandings of their CAM use are noticeably marked in ways that support their claims to normative masculine selves [36]. Whereas men might interpret the discourse of well-being as problematic, weakening their masculine image, women may experience the values embodied in the discourse of well-being in a different way [37]. This may further explain why more women in our study reported seeking CAM use in a need to improve their health and well-being.

Another finding from our analyses was that women who used CAM were more likely to report positive outcomes and greater benefit from their CAM use compared to male CAM users. Putting the potential social desirability bias aside, one possible explanation for this finding may be that women are more responsive to the effects of CAM on mind-body well-being, although this speculation has yet to be substantiated by future studies examining both self-reported clinical outcomes and some objective biomarkers. Another possible explanation for this finding could be that the health care needs of women, such as the desire for autonomy in health care decisions [38] and the need to be heard [39, 40], are possibly better served by CAM. In fact, these needs parallel the very reasons why people often turn to CAM, including the need to be listened to and cared for [41] and the desire to take a more active role in maintaining their own health [42]. Nevertheless, further research is still required to help tease out such features and motivations in greater depth.

### 4.3. Health Conditions

The findings from our study indicate no substantial differences in the top health conditions that male and female CAM users report. In line with previous NHIS reports [3, 4], musculoskeletal pain dominates the top health conditions for which our respondents used CAM, regardless of gender. Among both men and women, back pain/problems were the most commonly reported of all the conditions surveyed in NHIS 2012, with a higher percentage (32%) of male participants relative to female participants (23%) reporting CAM use for this condition. These findings are interesting given that women report back pain more often than men, particularly during middle age [43]. Numerous surveys have found that men tend to delay visiting a conventional doctor and consult less often, compared to women [44], a tendency that may be inherent in the construction of masculine identities and the specific social context in which men behave [44]. It is possible that the self-help strategies afforded by many CAM practices, such as yoga, align well with an individual's masculine ideal of power and control with men more inclined to perceive themselves as being able to manage their own health problems and not requiring the help of others.

### 4.4. Limitations

There are several limitations to our study. First of all, as all of questions in NHIS are self-reported and most of CAM questions were asked regarding the experience in the past 12 months, our study is subject to recall bias and social desirability bias. Secondly, because of the nature of the cross-sectional study design, our findings should be interpreted with caution and we cannot draw conclusions about possible causal pathways between two explored variables in our study. These limitations should be balanced against the strengths of the study, including the large sample size and representativeness of the US population.

## 5. Conclusion

This is the first known study in the USA that has sought to understand how male CAM users differ from female CAM users with respect to sociodemographic characteristics and perceived benefits of CAM use. Our paper provides foundation information regarding gender difference of CAM use and provides a platform for further in-depth examination of how and why males and females differ in their reasons for CAM use. Furthermore, our findings demonstrate that it is important that those in clinical practice engage and enquire with their male and female patients regarding possible CAM use in order to help provide safe, effective, coordinated, and equitable health care.

## Figures and Tables

**Table 1:** Weighted sociodemographic characteristics of non-CAM users and CAM users and male and female CAM users.

	Non-CAM user (*n* = 24344)%	CAM user (*n* = 10181)%	*P* value	CAM users		*P* value
				Male (*n* = 3907)%	Female (*n* = 6274)%	
Age, mean (95% CI)	46.5 (46.2, 46.8)	47.0 (46.5, 47.7)	0.070	47.3 (46.7, 47.9)	46.7 (46.2, 47.2)	0.174
Sex			<0.001			
Male	51.2	40.9				
Female	48.8	59.1				
Region of USA (*n* = 34,525)			<0.001			0.22
Northeast	18.7	17.0		15.9	17.7	
Midwest	21.5	25.6		25.3	25.8	
South	39.7	28.8		29.3	28.5	
West	20.1	28.6		29.6	28.0	
Race/ethnicity (*n* = 34,525)			<0.001			0.93
Hispanic	17.2	9.5		9.5	9.4	
Non-Hispanic White	62.8	77.4		77.4	77.4	
Non-Hispanic Black	14.0	6.8		6.9	6.8	
Non-Hispanic Asian	5.2	5.5		5.6	5.5	
Non-Hispanic Other	0.8	0.8		0.7	0.8	
Education			<0.001			0.001
Less than high school	20.6	8.3		9.8	7.3	
High school graduate and some degree	56.3	50.9		50.1	51.5	
Bachelor degree	15.7	24.7		23.2	25.7	
Master degree and higher	7.4	16.1		16.9	15.5	
Personal earning in the past year (US$)			<0.001			<0.001
<$10,000	8.1	8.2		6.7	9.2	
$10,000–$19,999	9.3	8.1		6.6	9.2	
$20,000–$34,999	11.8	12.6		11.9	13.1	
$35,000–$54,999	11.0	14.0		14.9	13.3	
$55,000–$74,999	5.3	8.9		10.4	7.9	
$75,000+	6.7	12.1		18.6	7.6	
Refused to report or do not know	11.4	9.0		9.1	8.9	
Did not work in the past year	36.4	27.1		21.7	30.9	
Marital status/relationship			<0.001			<0.001
Married or living with a partner	58.6	64.4		68.3	61.6	
Divorced or separated	11.2	11.5		9.6	12.8	
Widowed	6.5	4.9		1.9	6.9	
Never married	23.6	19.3		20.1	18.8	
Family spending on medical care			<0.001			0.12
0	12.9	7.0		7.6	6.6	
$1–499	35.9	29.2		28.0	30.0	
$500–1999	30.1	34.3		34.5	34.2	
$2000–2999	9.7	11.8		12.9	11.1	
$3000–4999	5.6	9.1		8.7	9.4	
$5000+	5.8	8.6		8.3	8.8	

**Table 2:** Weighted logistic regression models of CAM use^*^.

	Overall model				Men only				Women only			
	OR^†^	95% CI^‡^		*P*	OR	95% CI		*P*	OR	95% CI		*P*
Female^1^	2.75	2.50	3.03	0.00								
Region of USA												
Midwest^2^	1.43	1.24	1.65	0.00	1.19	0.94	1.50	0.16	1.69	1.39	2.05	0.00
South	0.70	0.60	0.82	0.00	0.87	0.70	1.09	0.24	0.63	0.52	0.78	0.00
West	2.87	2.46	3.35	0.00	1.78	1.41	2.26	0.00	3.21	2.61	3.93	0.00
Race/ethnicity												
Non-Hispanic White^3^	3.01	2.70	3.36	0.00	5.93	4.45	7.91	0.00	2.11	1.81	2.46	0.00
Non-Hispanic Black	0.70	0.58	0.84	0.00	3.03	2.11	4.33	0.00	0.43	0.34	0.54	0.00
Non-Hispanic Asian	0.14	0.11	0.18	0.00	0.44	0.25	0.76	0.00	0.88	0.65	1.19	0.40
Non-Hispanic Other	1.34	0.81	2.23	0.26	3.47	1.67	7.21	0.00	1.07	0.53	2.18	0.85
Education												
High school graduate and some degree^4^	1.15	1.03	1.30	0.02	2.60	1.93	3.50	0.00	0.89	0.76	1.04	0.13
Bachelor degree	1.93	1.65	2.26	0.00	4.00	2.88	5.55	0.00	1.46	1.19	1.81	0.00
Master degree and higher	5.33	4.49	6.34	0.00	5.58	3.93	7.91	0.00	5.91	4.64	7.53	0.00
Personal earning in the past year (US$)												
$10,000–$19,999^5^	1.07	0.85	1.36	0.56	0.91	0.61	1.36	0.65	1.00	0.75	1.34	0.99
$20,000–$34,999	1.39	1.12	1.72	0.00	1.13	0.80	1.59	0.49	1.19	0.90	1.57	0.22
$35,000–$54,999	0.76	0.61	0.94	0.01	0.50	0.35	0.71	0.00	1.19	0.89	1.58	0.24
$55,000–$74,999	1.66	1.29	2.14	0.00	1.02	0.70	1.48	0.92	1.52	1.06	2.18	0.02
$75,000+	1.51	1.19	1.93	0.00	1.03	0.73	1.45	0.88	1.23	0.83	1.80	0.30
Refused to report or do not know	2.30	1.87	2.82	0.00	0.68	0.47	0.97	0.04	3.71	2.81	4.89	0.00
Did not work in the past year	1.10	0.93	1.31	0.27	0.79	0.58	1.08	0.14	0.89	0.71	1.12	0.33
Marital status/relationship												
Divorced or separated^6^	1.57	1.39	1.76	0.00	1.11	0.91	1.36	0.30	2.06	1.75	2.41	0.00
Widowed	2.06	1.81	2.34	0.00	3.06	2.41	3.89	0.00	2.85	2.39	3.39	0.00
Never married	0.99	0.85	1.14	0.84	0.95	0.78	1.16	0.61	0.97	0.79	1.19	0.76
Family spending on medical care												
$1–499	0.71	0.62	0.81	0.00	1.50	1.17	1.93	0.00	0.50	0.42	0.60	0.00
$500–1999	0.61	0.52	0.71	0.00	0.98	0.75	1.28	0.90	0.68	0.56	0.84	0.00
$2000–2999	1.84	1.55	2.18	0.00	0.83	0.59	1.18	0.30	3.98	3.12	5.08	0.00
$3000–4999	1.07	0.84	1.37	0.58	1.79	1.24	2.60	0.00	0.96	0.71	1.31	0.81
$5000+^7^	0.92	0.73	1.17	0.51	1.29	0.90	1.86	0.17	1.01	0.73	1.38	0.97

^*^CAM use: use at least one CAM modality vs. no CAM use at all. ^†^OR: odds ratio; ^‡^CI: confidence interval.

^1^Reference = male; ^2^reference = Northeast; ^3^reference = Hispanics; ^4^reference = less than high school; ^5^reference = less than $10,000; ^6^reference = married or living together; ^7^reference = 0.

**Table 3:** Comparison of the reasons, motivations, and outcomes for using the first top CAM therapy by gender (*n* = 10,181).

Perceived benefits	Male (*n* = 3,880)%	Female (*n* = 6201)%	*P* value
Reasons			
For general wellness or general disease prevention	62.2	66.8	<0.001
To improve energy	28.4	38.5	<0.001
To improve immune function	23.2	28.4	<0.001
To improve athletic or sports performance	23.7	18.3	<0.001
To improve memory or concentration	16.0	18.2	0.024
Motivations			
To eat healthier	20.2	27.3	<0.001
To eat more organic foods	11.9	14.2	0.009
To cut back on or stop drinking alcohol (*n* = 7,082)^1^	7.0	7.1	0.873
To cut back on or stop smoking cigarettes (*n* = 1,509)^2^	13.3	15.1	0.427
To exercise more regularly	22.8	30.0	<0.001
Outcome			<0.001
Gave a sense of control over one's health	36.8	43.8	<0.001
Reduced stress level or relaxation	41.9	54.5	<0.001
Better sleep	36.1	43.4	<0.001
Feeling better emotionally	33.6	45.2	<0.001
Made it easier to cope with health problems	32.5	37.5	<0.001
Improved overall health and feeling better	67.3	71.1	0.001
Improved relationship with others	20.1	22.6	0.024
Improved attendance at job or school (*n* = 7514)^3^	16.2	16.8	0.562
How much the first therapy helped with the most important reasons for CAM use (*n* = 9,110)^4^			<0.001
A great deal	37.2	45.2	
Some	44.0	41.8	
Only a little	15.3	10.5	
Not at all	3.6	2.5	
Used first of top three therapies for specific health problems	43.0	42.9	0.924
How much the first therapy helped with specific health problems (*n* = 4,353)^4^			<0.001
A great deal	47.5	56.0	
Some	36.5	31.6	
Only a little	11.7	9.1	
Not at all	4.3	3.4	

^1^Sample adults 18+ who have used first of top three modalities and who have consumed alcohol in the past 12 months.

^2^Sample adults 18+ who have used first of top three modalities and who currently smoke every day or some days.

^3^Sample adults 18+ who have used first of top three modalities and who worked or attended school in the past year.

^4^Sample adults 18+ who have used first of top three modalities and two or more reasons for seeing a practitioner/using modality chosen.
